# α-Glucosidase Inhibitors from *Vauquelinia corymbosa*

**DOI:** 10.3390/molecules200815330

**Published:** 2015-08-21

**Authors:** Laura Flores-Bocanegra, Araceli Pérez-Vásquez, Mariana Torres-Piedra, Robert Bye, Edelmira Linares, Rachel Mata

**Affiliations:** 1 Facultad de Química, Universidad Nacional Autónoma de México, Mexico City 04510, Mexico; E-Mails: cecilau.gem@gmail.com (L.F.-B.); peva01@yahoo.com.mx (A.P.-V.); torrespiedra@gmail.com (M.T.-P.); 2 Instituto de Biología, Universidad Nacional Autónoma de México, Mexico City 04510, Mexico; E-Mails: rbyeunam@ib.unam.mx (R.B.); mazari@ib.unam.mx (E.L.)

**Keywords:** diabetes, yeast and rat α-glucosidases, *Vauquelinia corymbosa*

## Abstract

The α-glucosidase inhibitory activity of an aqueous extract and compounds from the aerial parts of *V. corymbosa* was demonstrated with yeast and rat small intestinal α-glucosidases. The aqueous extract inhibited yeast α-glucosidase with a half maximal inhibitory concentration (IC_50_) of 28.6 μg/mL. Bioassay-guided fractionation of the extract led to the isolation of several compounds, including one cyanogenic glycoside [prunasin (**1**)], five flavonoids [(−)-epi-catechin (**2**), hyperoside (**3**), isoquercetin (**4**), quercitrin (**5**) and quercetin-3-*O*-(6′′-benzoyl)-β-galactoside (**6**)] and two simple aromatic compounds [picein (**7**) and methylarbutin (**8**)]. The most active compound was **6** with IC_50_ values of 30 μM in the case of yeast α-glucosidase, and 437 μM in the case of the mammalian enzyme. According to the kinetic analyses performed with rat and yeast enzymes, this compound behaved as mixed-type inhibitor; the calculated inhibition constants (*K_i_*) were 212 and 50 μM, respectively. Molecular docking analyses with yeast and mammalian α-glucosidases revealed that compound **6** bind differently to these enzymes. Altogether, the results of this work suggest that preparations of *V. corymbosa* might delay glucose absorption *in vivo*.

## 1. Introduction

Type 2 diabetes mellitus (T2DM) is a chronic metabolic disorder whose prevalence has been increasing steadily all over the world [1]. In 2014, around 348 million people worldwide had T2DM, 77% of which lived in low- and middle-income countries [2,3]. Furthermore, the prevalence of T2DM in the next two decades will increment by 53% due to increase in ageing population [2,3]. The disease results from defects in insulin secretion, insulin action, or both, and it is characterized by a chronic hyperglycemia state [1].

The best treatment for T2DM is based on an adequate control of the hyperglycemia condition, which is possible to achieve with a healthy lifestyle and appropriate pharmacological therapies [4]. These treatments include drugs that promote insulin secretion (sulfonylureas) or utilization (biguanides and thiazolidinediones) and, drugs that reduce the rate of carbohydrate absorption from the gastrointestinal tract and decrease postprandial glucose peak [4]. The latter group includes α-glucosidase inhibitors, of which acarbose and miglitol are the most widely used [5,6]. In recent years substantial efforts have been made to discover new natural effective α-glucosidase inhibitors useful for the development of new remedies; in this regard medicinal plants have shown to be valuable sources of these inhibitors [7].

In contemporary Mexico, an infusion of *Vauquelinia corymbosa* Bonlp (Rosaceae) is regarded as useful for the treatment of diabetes [8,9]. Therefore, this species was considered as potential source of α-glucosidase inhibitors suitable for the development of new antidiabetic drugs. Previous phytochemical studies of the aerial parts of *V. corymbosa* resulted in the isolation and characterization of three triterpenoids, namely ursolic and betulinic acids as well as uvaol [10]. 

## 2. Results and Discussion

### 2.1. Isolation of α-Glucosidase Inhibitors from V. corymbosa

**Figure 1:**
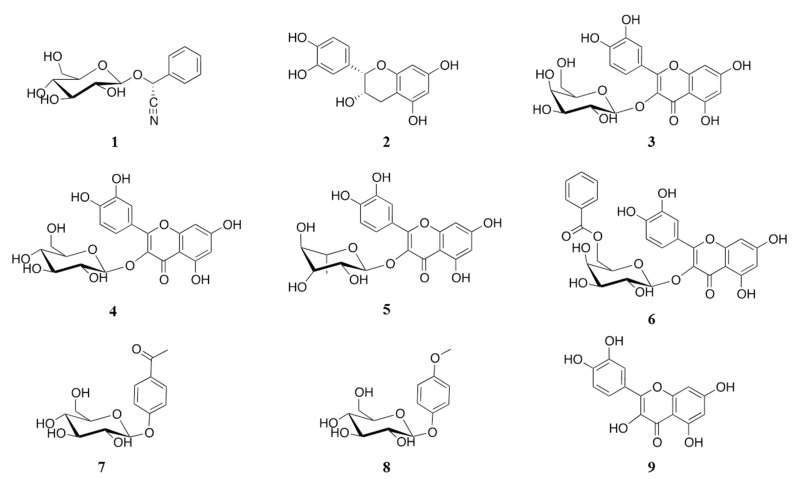
Structure of compounds **1**–**8** isolated from *V. corymbosa* and quercetin (**9**).

An aqueous extract (AE) of *V. corymbosa* inhibited in a concentration-dependent manner (IC_50_ = 28.5 μg/mL) the enzymatic activity of yeast α-glucosidase when tested by a well-known spectrophotocolorimetric assay [11]. This result suggested the presence in AE of active compounds with potential antihyperglycemic properties. Fractionation of the active extract led to the isolation of one cyanogenic glycoside [prunasin (**1**) [12,13]], several flavonoids [(−)-*epi*-catechin (**2**) [14], hyperoside (**3**) [15], isoquercetin (**4**) [16,17], quercitrin (**5**) [18], and quercetin-3-*O*-(6′′-benzoyl)-β-galactoside (**6**) [19]] and two simple aromatic compounds [picein (**7**) [20] and methylarbutin (**8**) [21]]. Compounds **1**–**8** (Figure 1) were identified by comparison of their spectroscopic and spectrometric data with those previously described. Compound **6** belongs to a rare group of flavonoid glycosides benzoylated at some point of the sugar moiety; this is the second report of its detection in nature.

All isolates were tested against yeast and mammalian α-glucosidases; acarbose and compounds **2**–**4**, which are well-characterized α-glucosidase inhibitors [22,23,24], were used as positive control. The most effective compounds against both enzymes were **4** and **6** (Table 1). The better activity of compound **6** in comparison with **3** suggested that benzoylation at C-6′′ of the sugar moiety increased notably the inhibitory activity against both enzymes. On the other hand, the nature of the sugar seems to have an impact on the inhibitory action *in vitro* since the glucoside **4** was more active than the galactoside **3** and rhamnoside **5**. Although flavonoids are well known α-glucosidase inhibitors, this is the first report regarding the α-glucosidase inhibitory properties of a cyanogenic glucoside, which as catechin (**2**) was more active against the yeast enzyme. On the contrary, picein (**7**) was more active against the rat mixture of enzymes. In any case, the active extract of *V. corymbosa* contains compounds capable of inhibiting yeast, and mammalian α-glucosidases. The effect on the latter enzyme correlated well with the potential antihyperglycemic action of this species.

**Table 1:** Effect of compounds **1**–**9** on the enzymatic activity of yeast and rat small intestinal α-glucosidases.

Sample	Yeast		Rat Small Intestinal	
	IC_50_ (mM) ^a^	Maximum Inhibition (%)	IC_50_ (mM) ^a^	Maximum Inhibition (%)
Acarbose ^b^	0.50 ± 0.23	89.7	0.10 ± 0.003	80.2
**1**	1.60 ± 0.07	95.2	ND	44.5 ^d^
**2** ^b^	0.30 ± 0.02	99.6	ND	15.2 ^d^
**3** ^b^	0.40 ± 0.02	67.8	1.98 ± 0.15	65.1
**4** ^b^	0.06 ± 0.005	99.0	1.63 ± 0.11	71.0
**5**	ND ^c^	45.0	3.34 ± 0.38	59.7
**6**	0.03 ± 0.006	95.6	0.43 ± 0.03	75.8
**7**	Inactive	-	10.68 ± 0.96	52.6
**8**	Inactive	-	ND	39.3 ^d^
**9**	0.03 ^e^	ND	0.216 ^e^	ND

^a^ Values present mean ± SD of triplicate experiments; ^b^ Positive controls; ^c^ Non-determined; ^d^ % Inhibition obtained with 10.0 mM of inhibitor; ^e^ values taken from reference [22].

It is interesting to point out, that infusions or decoctions from other plants containing cyanogenic glycosides such as *Eriobotrya japonica* and *Prunus amygdalus* are used also as antidiabetic agents in several Eastern countries [25,26]. However, animal studies conducted with amygdalin and other cyanogenic did not revealed hypoglycemic action [25]. Therefore, it is highly probable that amygdalin and congeners also inhibit the activity of α-glucosidases. The presence in *V. corymbosa* of compounds **3**–**5** could have important implications on the *in vivo* activity of the plant, since upon consumption of its phytopreparations quercetin could be readily generated to exert its well-known hypoglycemic and α-glucosidase inhibitory activities [27,28].

### 2.2. Mode of Inhibition of Yeast and Mammalian α-Glucosidases and Molecular Docking for Compound **6**

**Figure 2:**
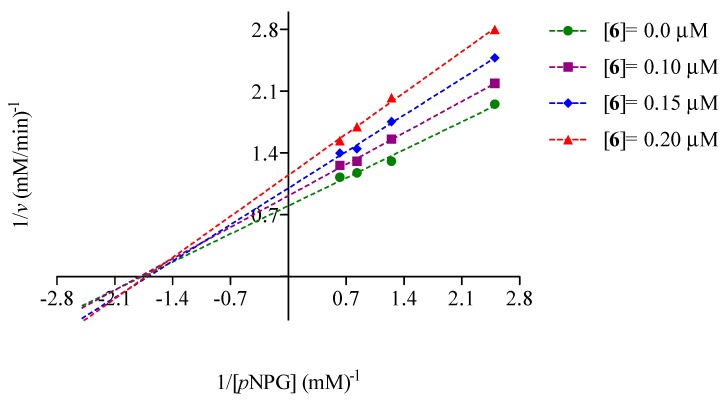
Lineweaver-Burk plot of yeast α-glucosidase inhibition of **6**.

**Figure 3:**
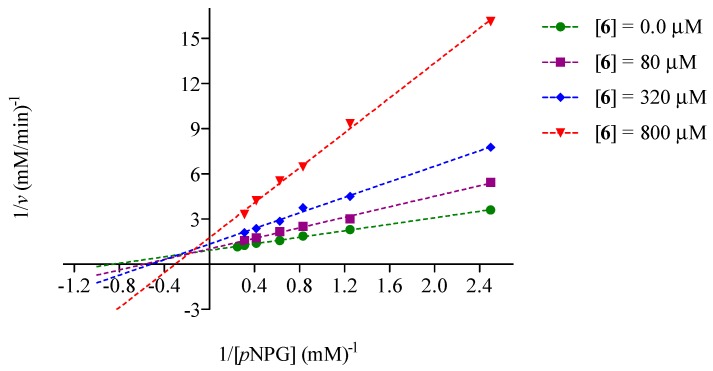
Lineweaver-Burk plot of small intestinal rat α-glucosidase inhibition of **6**.

Kinetic analysis of **6** revealed that it behaved as a mixed type inhibitor, which is common among natural products [29,30]. Figure 2 and Figure 3 show its Lineweaver-Burk plots; the calculated *K_i_* values were 0.05 ± 0.02 mM (R^2^ = 0.9925) for yeast α-glucosidase and 0.212 ± 0.031 mM (R^2^ = 0.9944) for the mammalian enzyme. Thus, compound **6** can bind both the free enzymes and the enzyme-substrate complexes.

**Figure 4:**
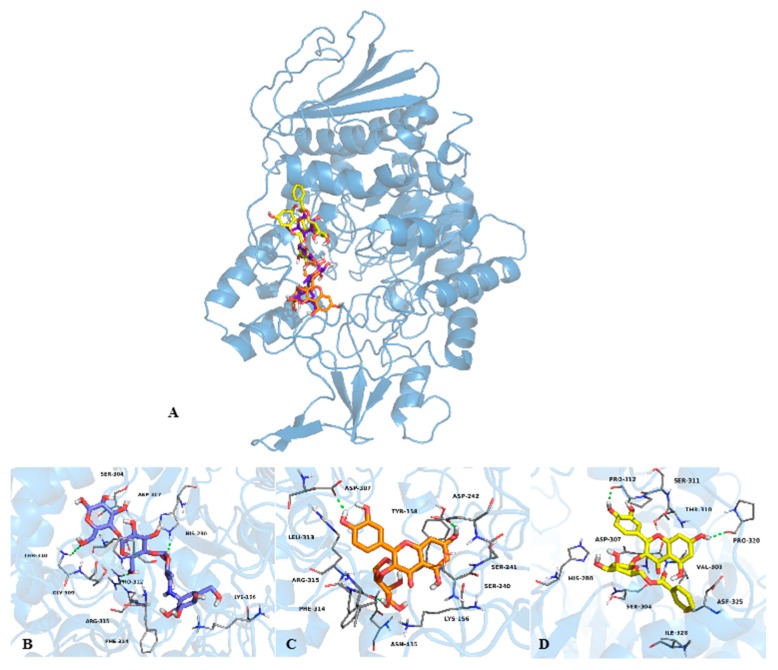
(**A**) Structural model of the complex acarbose (purple sticks), **4** (orange sticks) and **6** (yellow sticks) with yeast α-glucosidase; 3D representation of the interaction between acarbose (**B**); **4** (**C**) and **6** (**D**) with the enzyme in the predicted binding site.

**Figure 5:**
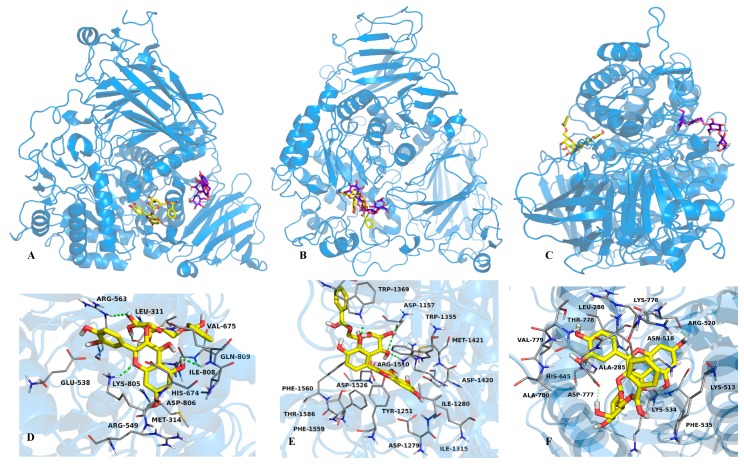
Structural models of the complexes acarbose (purple sticks) and **6** (yellow sticks) with 3LPP (**A**), 3TOP (**B**) and 2QMJ (**C**) enzymes; 3D representation of the interactions between **6** and the enzymes 3LPP (**D**); 3TOP (**E**) and 2QMJ (**F**), in the predicted binding sites.

In order to predict the binding mode of compound **6** to yeast and mammalian α-glucosidases, docking analyses with yeast isomaltase, human N-terminal sucrase isomaltase (3LPP), human N-terminal maltase glucoamylase (2QMJ) and human C-terminal maltase glucoamylase (3TOP) were performed. The human enzymes were selected because its similarity to the rat small intestinal enzymes. In general, the ligands were docked into the entire enzyme, and the best conformations observed in this preliminary study were docked into a smaller area, in order to refine the results (Figure 4 and Figure 5).

The docking study with compound **6** predicted that it binds to yeast isomaltase near to the catalytic site, forming two hydrogen bonds with Pro312 and Pro320 through the hydroxyl groups at C-2′ and C-7 respectively; other interactions involved Van der Waals forces with His280, Ser311, Thr310, Val308, Ser304, Asp325, Ile328 and Asp307 (Figure 4D). On the other hand, compound **3**, which was included for comparative purpose, bind at the catalytic site, forming hydrogen bonds between Asp307, Leu313 and Asp242 and the hydroxyl groups at C-4′, C-2′′ and C-7, respectively; Van der Waals forces interactions were observed throughout Phe314, Asn415, Lys156, Ser240, Tyr158, Ser241 and Arg315 (Figure 4C). Compounds **3** and **6** bind to yeast isomaltase similarly to other flavonoids, with their B rings embedded deep inside the binding cavity and, rings A and C flanking out (Suplementary Data Figures S15 and S16) [31]. The analysis with 3TOP predicted that **6** posed in the same site of acarbose forming four hydrogen bonds between the hydroxyl groups at C-3′, C3′′ and C-2′′, and the aminoacids Trp1369, Arg1510 and Asp1157; Van der Waals interactions between **6** and the aminoacids Asp1526, Phe1560, Phe1559, Met1421, Trp1355, Asp1420, Try1251, Thr1586, Ile1315, Ile1280 and Asp 1279 were also observed (Figure 5, panels B and E). With 2QMJ (Figure 5, panels C and F), compound **6** bind in a site far away from that of acarbose, and formed four hydrogen bonds between the hydroxyl groups at C-5, C-7, C4′ and C-4′′, and the aminoacids Lys534, Arg520, Thr778 and Ala780; Van der Waals interactions were established with Lys513, Phe535, Asn518, Lys776, Asp777, Leu286, Ala285, Val779 and His645. Finally, flavonol **6** interacted with 3LPP (Figure 5, panels A and D) in a place close to the catalytic site. The compound formed also four hydrogen bonds between the hydroxyl groups at C-5, C-2′′, C3′′ and the oxygen of the γ-pyran moiety, and the aminoacids Ile808, Arg563, Leu311 and Lys805; Van der Waals interactions were established with Glu538, His569, Lys805, Met314, Arg549, His674, Gln809, Val675, Asp806 and 563.

The molecular docking analyses with yeast and mammalian α-glucosidases revealed that compound **6** bind differently to these enzymes. The calculated binding constants (*K_i_*) revealed also different affinities. Thus, compound **6** showed the smallest inhibition constant for the N-terminal maltase glucoamylase (*K_i_* = 70.6 nM), followed for the C-terminal maltase glucoamylase (*K_i_* = 133.1 µM), the N-terminal sucrase isomaltase (*K_i_* = 2.3 mM), and finally for the yeast isomaltase (*K_i_* = 42.6 mM). These results contrasted with the experimental results since compound **6** was more active against the yeast isomaltase than against the mixture of rat intestinal enzymes. Kinetic analysis with purified individual proteins will provide conclusive idea about compound **6**’s α-glucosidase binding preference.

### 2.3. Determination of Acute Toxicity Studies of Some Extracts of V. corymbosa

The presence of prunasin, a cyanogenetic glycoside and the fact that this species is consumed in folk medicine practices prompted us to assess the potential toxic effects of AE and an organic extract (OE) from *V. corymbosa*. For this endeavor the Lorke method was employed [32]. In the case of AE, no animal death was recorded during the two phases of the test, and no damage of internal organs was recorded on macroscopic inspection. The median lethal dose (LD_50_) for AE was estimated to be higher than 5 g/kg. However, OE caused the typical signs of cyanosis since purple coloration was observed in upper and lower limbs, ears and nose; however, no appreciable damage of internal tissues or main organs was detected; the calculated LD_50_ was 2.6 g/kg. These results suggested a higher content of prunasin in OE (0.23% in OE *vs.* 0.05% in AE). In this regard, it is well known that enzymatic hydrolysis of cyanogenic glycosides is enhanced by several factors, including temperature; thus, during preparation of the infusion with boiling water prunasin could have been hydrolyzed to benzaldehyde and the highly toxic hydrogen cyanide (HCN), which volatilized [33,34]. Accordingly, HPLC analysis of the infusion (Supplementary Data Figure S1) showed that **1** was one of the minor compounds detectable in this preparation. The remaining isolated compounds were also identified in the resulting representative chromatogram of the infusion (Supplementary Data Figure S1). Although AE did not produce significant acute toxic effects in mice, it is important to alert consumers about the risk of the long-term use of this medicinal plant. 

## 3. Experimental Section 

### 3.1. General Procedures

NMR spectra [400 MHz (^1^H-NMR) or 100 (^13^C-NMR)] including COSY, HMBC and HSQC experiments were recorded in CH_3_OH-*d*_4_ or DMSO-*d*_6_ solution on a Unity Inova 400 spectrophotometer (Varian, Palo Alto, CA, USA) using tetramethylsilane (TMS) as internal standard. HPLC analyses were carried out in a Waters HPLC instrument equipped with a Waters 996 UV photodiode array detector (900) set at 205–400 nm (Waters, Milford, MA, USA). Control of equipment, data acquisition processing and management of chromatographic information were performed by the Empower 2 software package (Waters). TLC analyses were performed on silica gel 60 F_254_ plates (Merck, Kenilworth, NJ, USA), and visualization of plates was carried out using a Ce_2_(SO_4_)_3_ (10%) solution in H_2_SO_4_. For open column chromatography (OCC), Silica gel 60 (0.063–0.200 mm), 70–230 mesh (Merck), or Sephadex^®^ LH-20 (Sigma-Aldrich-Fluka, St. Louis, MO, USA) was employed. 

### 3.2. Plant Material

Branches and leaves of *V. corymbosa* were collected on 26 February 2012, in San Borja Chihuahua, Mexico. The plant was identified by R. Bye and E. Linares. A voucher specimen (R. Bye and E. Linares 36951) has been deposited at the National Herbarium (MEXU), UNAM, Mexico City, Mexico.

### 3.3. Preparations of Extracts

Dried and ground aerial parts (500 g) were extracted with 14 L of boiling water during 30 min, then filtered and dried to yield an aqueous extract (AE, 14.0 g). The infusion was extracted with ethyl acetate (EtOAc, 3 × 14 L) to produce two fractions, namely EtOAc-soluble (ESF, 5.5 g) and H_2_O-soluble fractions (WSF, 8.5 g). ESF and WSF were tested in the enzymatic assay; ESF was the most effective, inhibiting the activity of the yeast enzyme by 92% at the concentration of 100 µg/mL and that of the rat small intestine by 50% at the same concentration. The organic extract (OE) was prepared by macerating dried and ground aerial parts (50 g) with a solvent mixture of CH_2_Cl_2_/CH_3_OH (1:1) during 15 days; after filtering, the extract was dried *in vacuo* to yield a brown residue (OE, 10.7 g).

### 3.4. Isolation of the Active Compounds from the Active EtOAc-Soluble Fraction

ESF (5.5 g) was dissolved in CH_3_OH and then separated by Sephadex chromatography to give ten secondary fractions (ESF1–ESF10) of which ESF3 and ESF6 were active. These two fractions, along inactive fractions ESF2, ESF4 and ESF7 were next chemically investigated. ESF3 (363 mg) was dissolved in CH_3_OH; after two h, 250 mg of **1** (0.05% of dry wt of plant material) spontaneously crystallized. ESF6 (85.0 mg) was separated by HPLC (SymmetryPrep RP-C8, CH_3_CN/H_2_O 20:80, 2.6 mL/min) to give 15 mg of **3**, 5 mg of **4**, 8 mg of **5** and 50 mg of **6** (R_t_ = 5.0, 5.5, 6.6 and 16.4 min, respectively). ESF2 (639 mg) was dissolved in CH_2_Cl_2_/CH_3_OH (1:1), from this solution crystallized 560 mg of **7**. ESF4 (119 mg) was further chromatographed on a silica gel (100 g) column, eluting with hexane with increasing amounts of EtOAc (50:50→0:100), and then with a mixture of increasing the polarity of EtOAc/CH_3_OH (0:100→80:20); altogether, this process yielded four secondary fractions (EFS4-1 EFS4-4); from fraction EFS4-3, eluted with hexane/EtOAc (8:2), 5 mg of compound **8** were obtained. EFS7 (580 mg) was dissolved in CH_3_OH and column chromatographed on Sephadex to yield 210 mg of compound **2**.

### 3.5. α-Glucosidase Inhibitory Assays 

AE, ESF, WSF, acarbose and compounds (**1**–**8**) were dissolved in CH_3_OH or phosphate buffer solution (PBS, 100 mM, pH = 7). Aliquots of 0–10 μL of testing materials (triplicated) were incubated for 10 min with 20 μL of an enzyme solution of yeast α-glucosidase (Sigma-Aldrich, 0.4 units/mL in PBS) or mammalian α-glucosidase (Sigma-Aldrich, 40 mg). The mammalian enzyme was separated from rat intestinal acetone powder, which was hand homogenized using 1 mL of ice-cold PBS. After sonicating during ten min, the content was centrifuged at 6400 rpm for 10 min (Microfuge Qualitron, Inc., Albany, NY, USA). The supernatant was recovered and used for the *in vitro* assay.

After incubation with the enzymes 10 μL of *p*-nitrophenyl α-d-glucopyranoside (*p*NPG 5 mM and 10 mM in PSB, respectively) were added and then incubated for 20 min at 37 °C. Then, absorbances were determined in a BIO-RAD microplate reader model 680 at 405 nm [11].

The concentration required to inhibit activity of the enzyme by 50% (IC_50_) was calculated by regression analysis, using the following equation [35]:

(1) $$\%Inhibition=A_{100}/1+{\left(I/{\text{IC}}_{50}\right)}^{S}$$

where, *A*_100_ is the maximum inhibition, *I* is inhibitor concentration and *s* is the degree of cooperation.

### 3.6. Kinetic Analyses

Mode of inhibition of compound **6** against yeast/mammalian α-glucosidase activity was measured with increasing concentrations of pNPG (0.4, 0.8, 1.2, 1.6 and 2.0 mM/0.4, 0.8, 1.2, 1.6, 2.4, 3.2, 3.6 and 4.0 mM, respectively) as a substrate, in the absence and presence of **6** at 0.1, 0.15 and 0.2 µM for yeast enzyme and 80, 320 and 800 µM for the mammalian enzyme.

The mode of inhibition was determined by the Lineweaver-Burk plots. The inhibition constants were calculated using the following equation [36]:

(2) $$v=V_{\text{max}}\times S/K_{m}\left(1+[I]/K_{i}\right)+S\left(1+[I]/\text{α}K_{i}\right)$$

where *v* is the initial velocity in absence and presence of inhibitor; *S* and *I* are concentration of substrate and inhibitor, respectively; *V*_max_ is the maximum velocity; *K_m_* is the Michaellis-Menten constant; *K_i_* is the competitive inhibition constant and α*K_i_* is the uncompetitive inhibition constant. The kinetic data was analyzed using a computer program for nonlinear regressions (Graph Pad Prism 5.0, GraphPad Software, Inc., La Jolla, CA, USA).

### 3.7. Docking Studies

Yeast isomaltase (PDB:3A4A), N-terminal sucrose isomaltase (PDB:3LPP), N-terminal maltase glucoamylase (PDB:2QMJ) and C-terminal maltase glucoamylase (PDB:3TOP) were used in this study as molecular target and crystallographic structures were downloaded from the Protein Data Bank (PDB). All hydrogen and Kolleman charges were assigned to receptors using AutoDockTools 1.4.5 with a semi-empirical mathematical method (PM3). The ligands (**3**, **6** and acarbose) were built using Spartan 2.0 and optimized geometrically with HyperChem 8.0; the ligands were prepared by assigning the atomic charges and nonpolar hydrogens using AutoDockTools 1.5.4 [37]. Docking studies were done with Lamarckian Genetic Algorithm (LGA) using the program AutoDock4.0 [38].Grid box for docking was set around central atom of the ligand with dimension of 126 × 126 × 126 Å in the *x*, *y* and *z* dimensions. The results were analyzed with AutoDockTools 1.5.4 [37] and PyMOL [39]. 

### 3.8. Isolation of Compound **1** from OE of V. corymbosa

OE (10.7 g) was separated by chromatography on a silica gel column, eluting with hexane with increasing amounts of EtOAc (10:0→7:3); this process yielded three secondary fractions (OE1–OE3); fraction OE2 (250 mg, eluted with hexane/EtOAc (8.5:1.5), afforded 115 mg of compound **1** (0.23% of dry wt of plant material). This activity was performed in order to compare the yields of **1** in aqueous and organic extracts.

### 3.9. Chromatographic Techniques 

The chromatographic profile of AE was obtained by reverse-phase HPLC (Symmetry C18 column, 5 µm, 4.6 mm internal diameter × 150 mm) using a as mobile phase CH_3_CN/H_2_O (acidified with 0.05% of CH_3_COOH) 2:8, and increasing linearly to 5:5 over 20 min; the latter conditions were held for 5 min, and then changed to 2:8 for 5 min and holding during 15 min. The flow rate was 0.6 mL/min and the injection volume was 20 μL.

### 3.10. Determination of Acute Toxicity for AE and OE 

Experiments were performed on male mice ICR (body weight, 25–30 g), obtained from Centro UNAM/Harlan (Harlan Mexico, S.A. de C.V., Mexico City, Mexico). All experiments were performed following the Mexican Official Norm for Animal Care and Handing (NOM-062-ZOO-1999). Mice were housed in a climate and light controlled room with a 12 h light/dark cycle. Four h before experiments, food was withheld, but animals had free access to drinking water. The extracts (AE and OE) were suspended in vehicle (saline solution 0.9%). The concentrations were adjusted to orally administrate 0.2 mL/10 g of body weight. Mice were treated in two phases: In the first one, doses of 10, 100 and 1000 mg/kg (*n* = 3 per dose) and, in the second, doses of 1600, 2900 and 5000 mg/kg (*n* = 3 per dose) of extracts were administered. In both stages, mice were observed daily in a period of 14 days for mortality, toxic effects and/or changes in behavioral pattern [32].

## 4. Conclusions 

The information generated in this study indicates that *V. corymbosa* is a source of new α-glucosidase inhibitors. Compound **6** showed good potential for the development of new chemical entities useful for the treatment of diabetes. Further *in vivo* studies are necessary to demonstrate its antihyperglycemic action. On the other hand, the presence in the plant of compounds **3**–**5** with known antidiabetic properties in animal models [40,41] might explain the reputed antidiabetic activity of *V. corymbosa* in Mexican folk medicine. Health Authorities should inform consumers about the risks of inappropriate usage of this species since it contains large amounts of cyanogenic glucosides.

## Supplementary Materials

Supplementary materials can be accessed at: http://www.mdpi.com/1420-3049/20/08/15330/s1.
