# Supplementary material for: α-Glucosidase Inhibitors from Vauquelinia corymbosa
Source: Molecules. 2015 Aug 21;20(8):15330–42. doi: 10.3390/molecules200815330 (PMC6332183; doi:10.3390/molecules200815330)
Supplement: Supplementary file 1 [file molecules-20-15330-s001.pdf]

Supplementary Materials

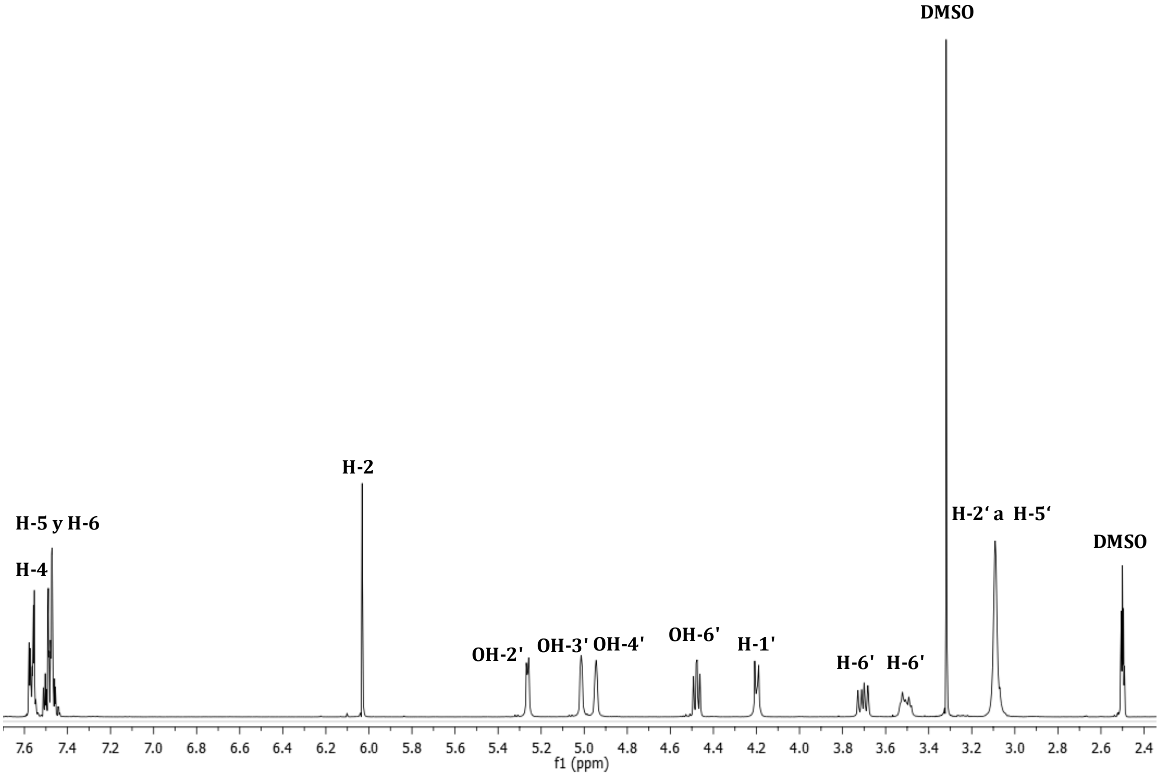

Figure S1. ^{1}H-NMR spectra of prunasin (**1**) (400 Hz, DMSO-*d*$_6$).

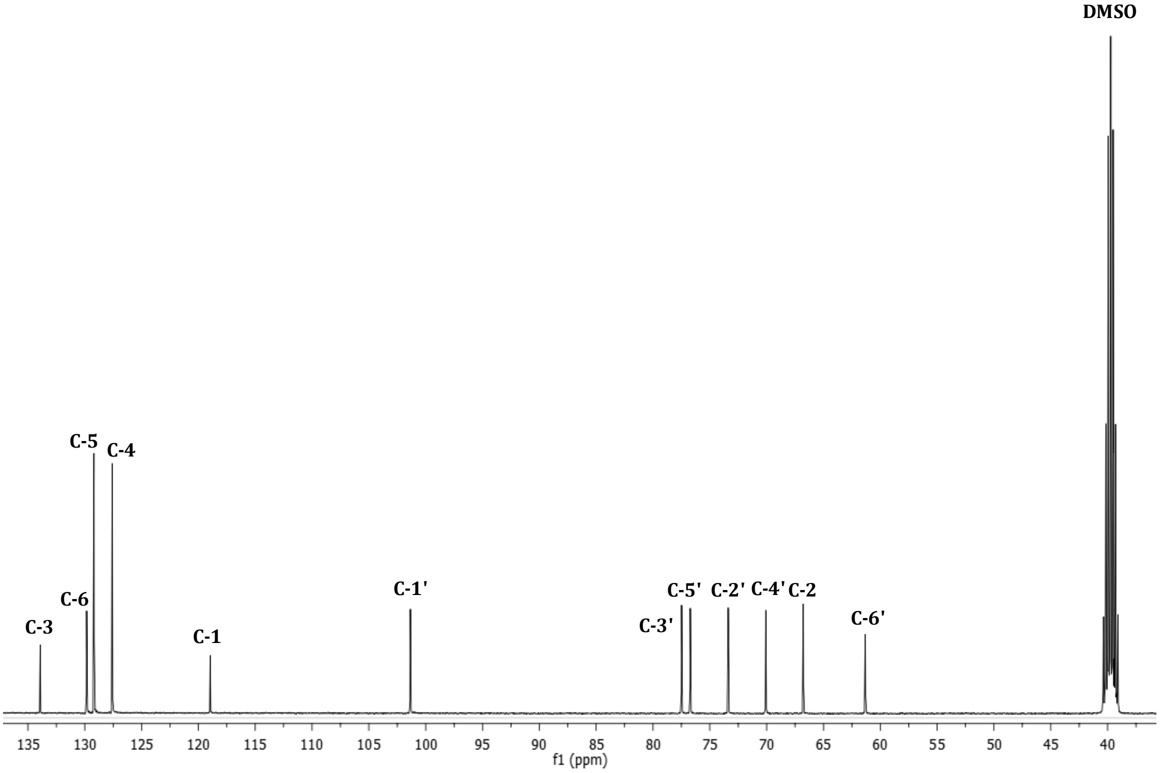

Figure S2. ^{13}C-NMR spectra of prunasin (**1**) (100 Hz, DMSO-*d*$_6$).

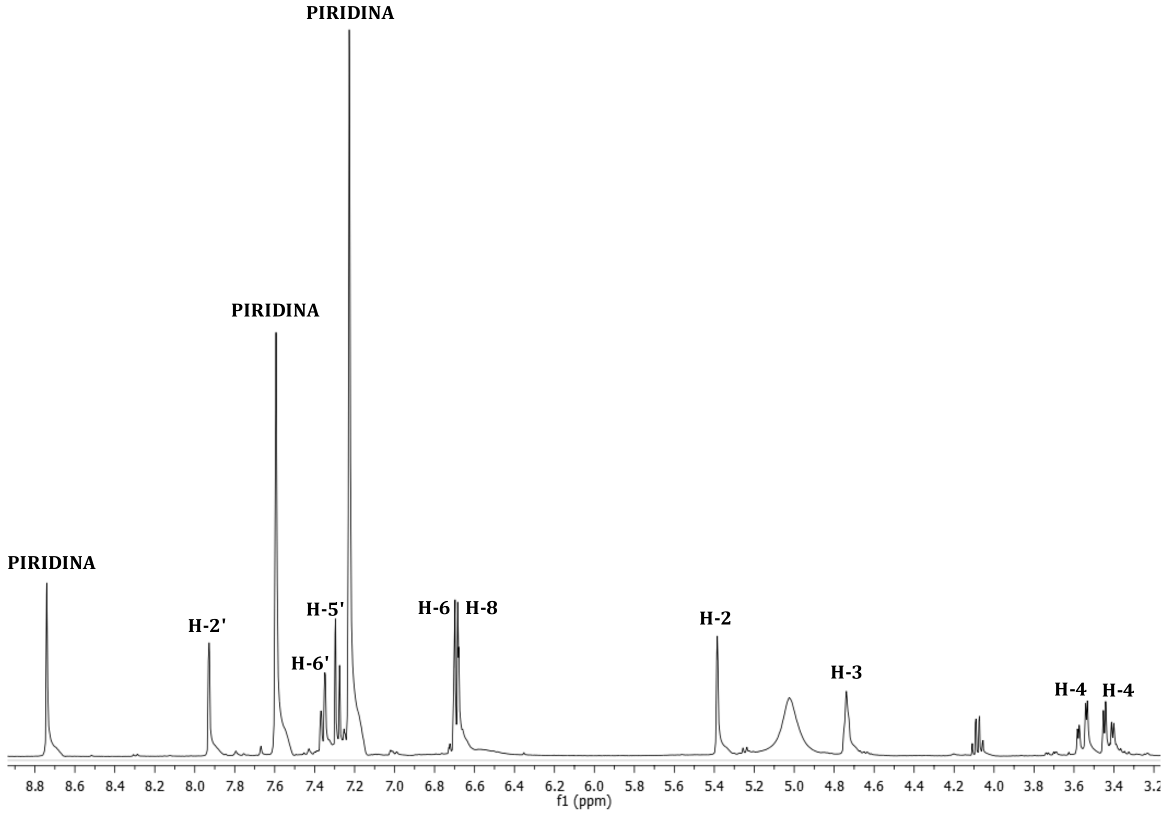

Figure S3. ^{1}H-NMR spectra of (−)-*epi*-catechin (**2**) (400 Hz, DMSO-*d*6).

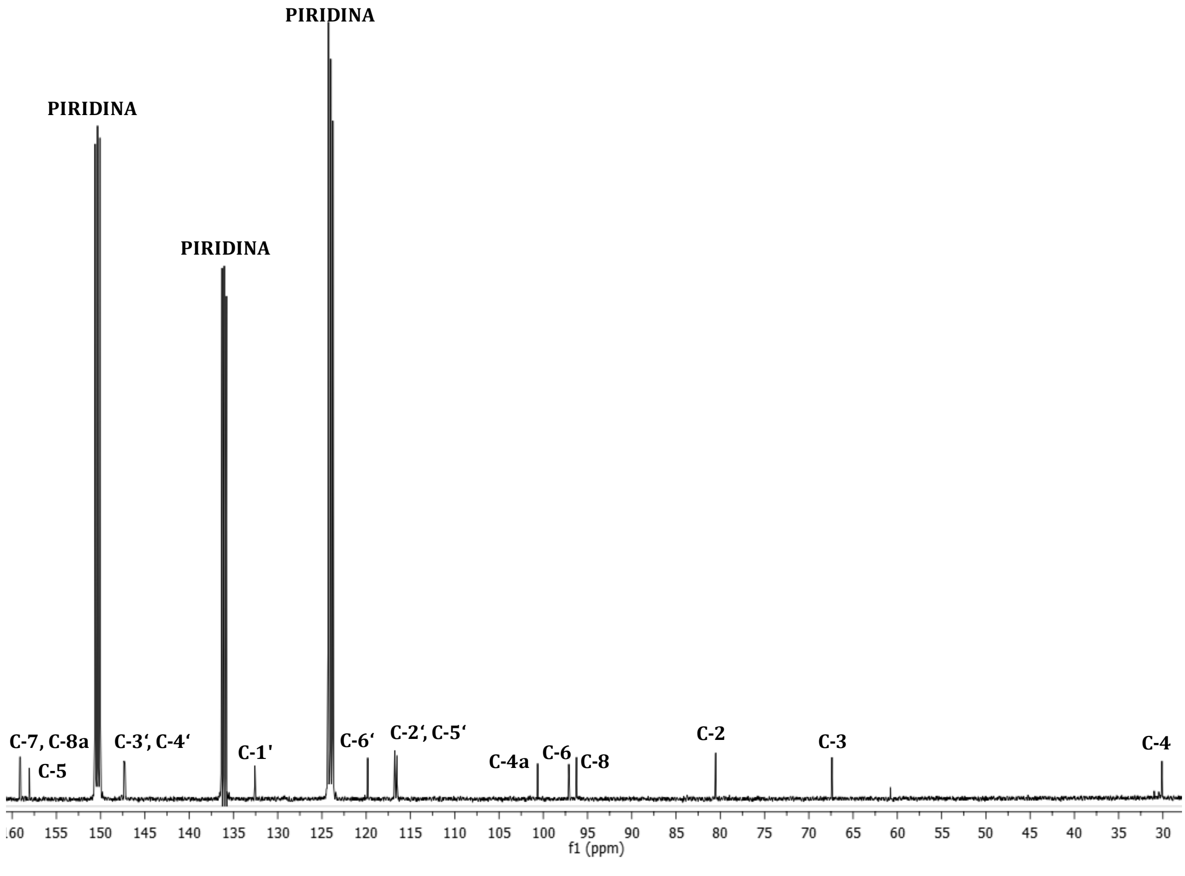

Figure S4. ^{13}C-NMR spectra of (−)-*epi*-catechin (**2**) (100 Hz, DMSO-*d*6).

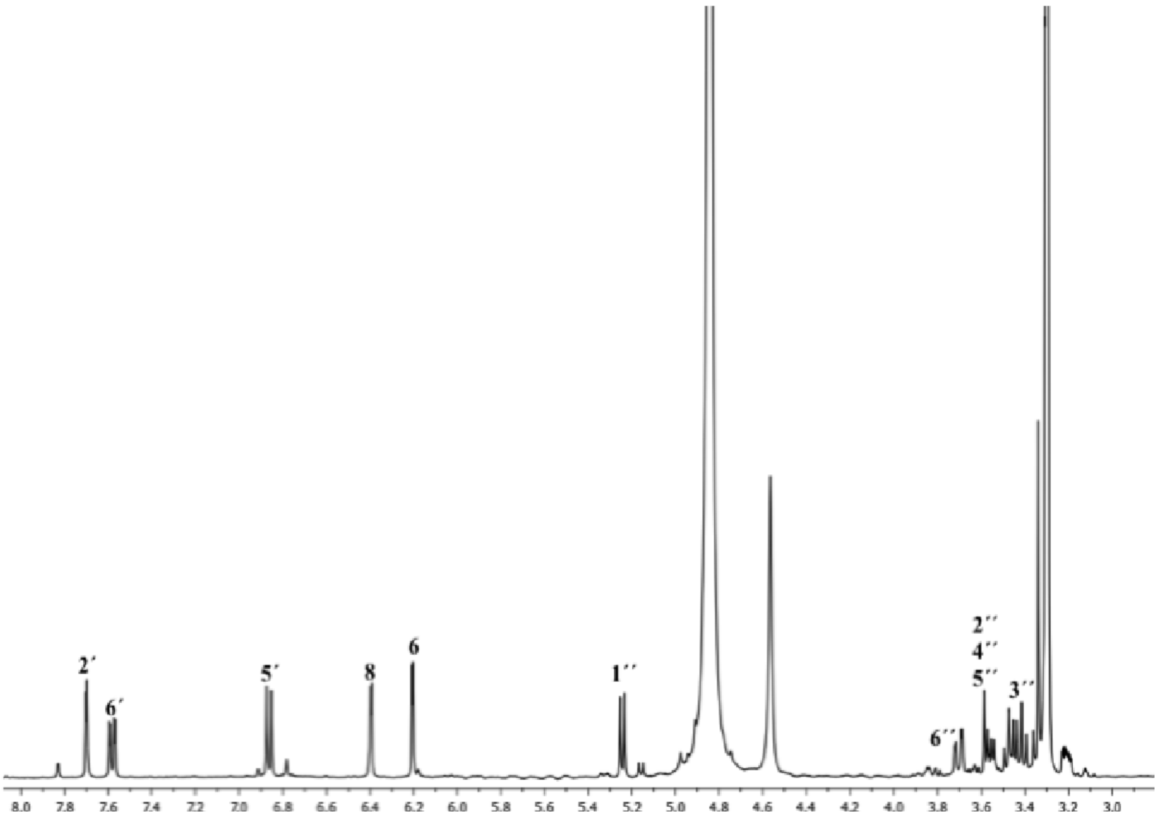

Figure S5. ^{1}H-NMR spectra of isoquercetin (**4**) (400 Hz, DMSO-d_6).

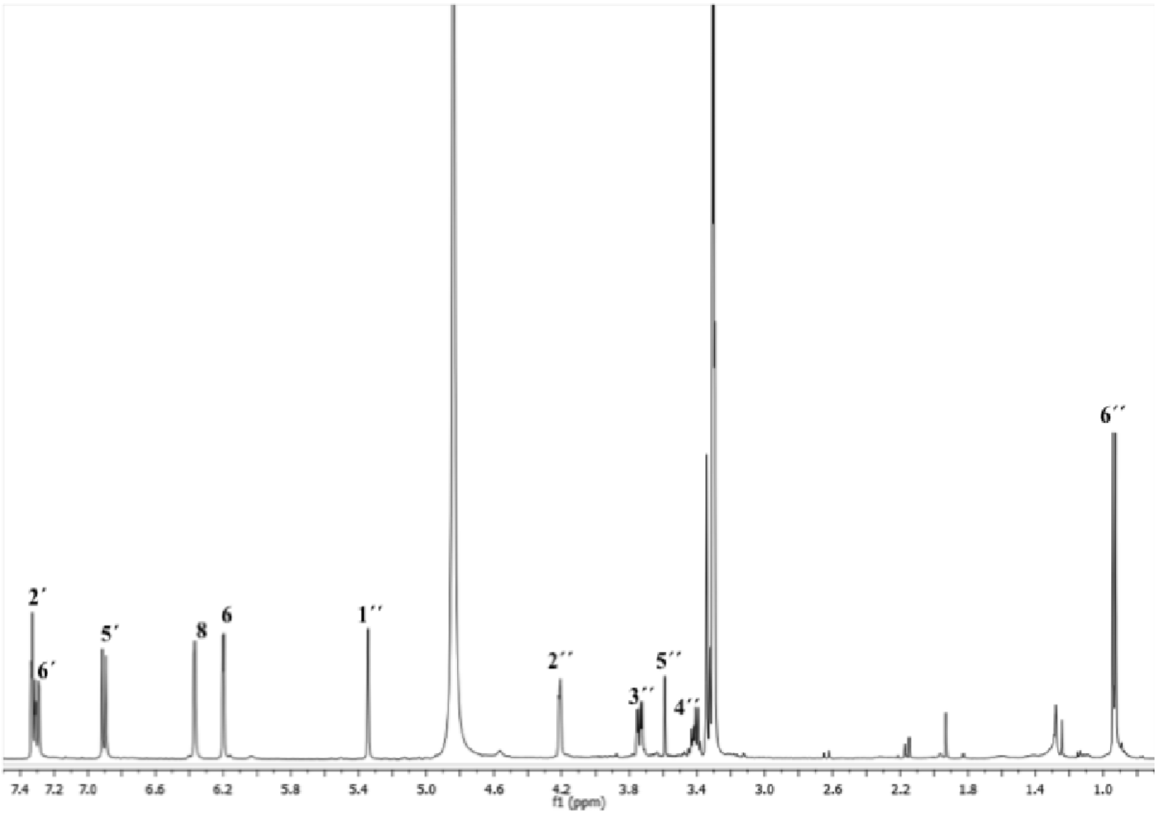

Figure S6. ^{1}H-NMR spectra of quercitrin (**5**) (400 Hz, DMSO-d_6).

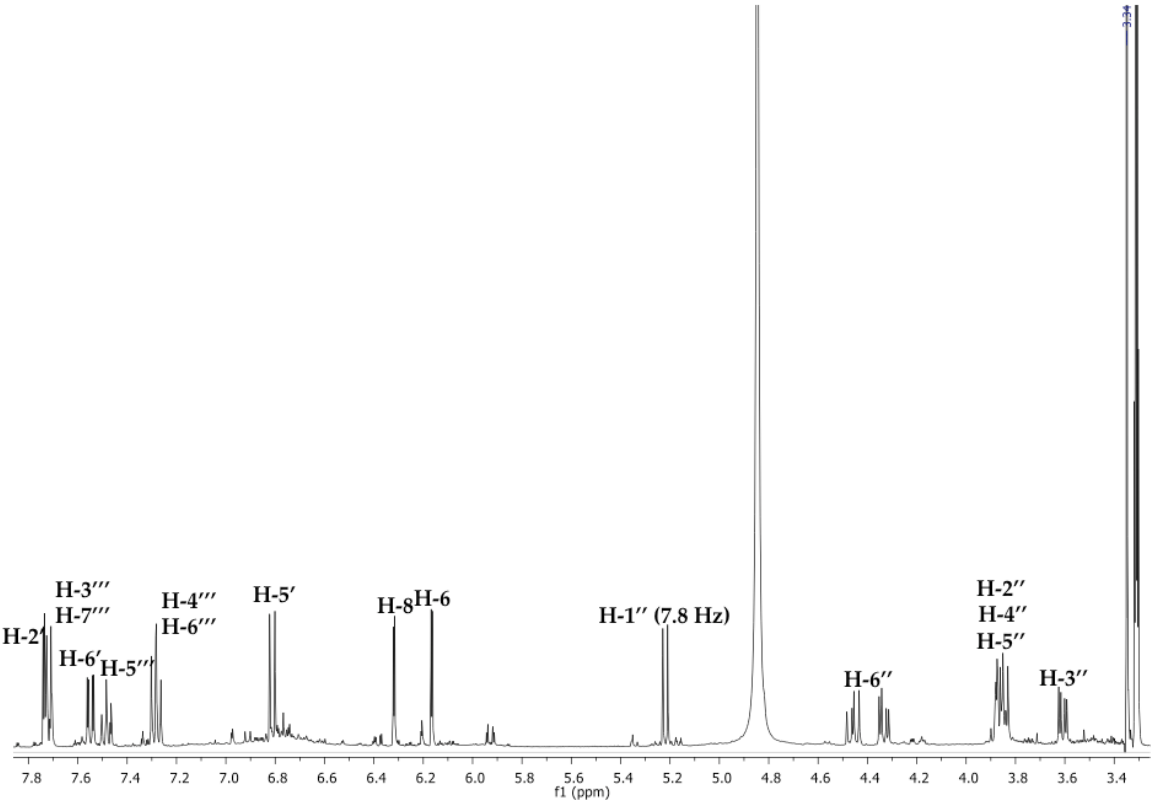

Figure S7. ^{1}H-NMR spectra of quercetin-3-*O*-(6″-benzoyl)-β-galactoside (**6**) (400 Hz, CH_3OH-d_4).

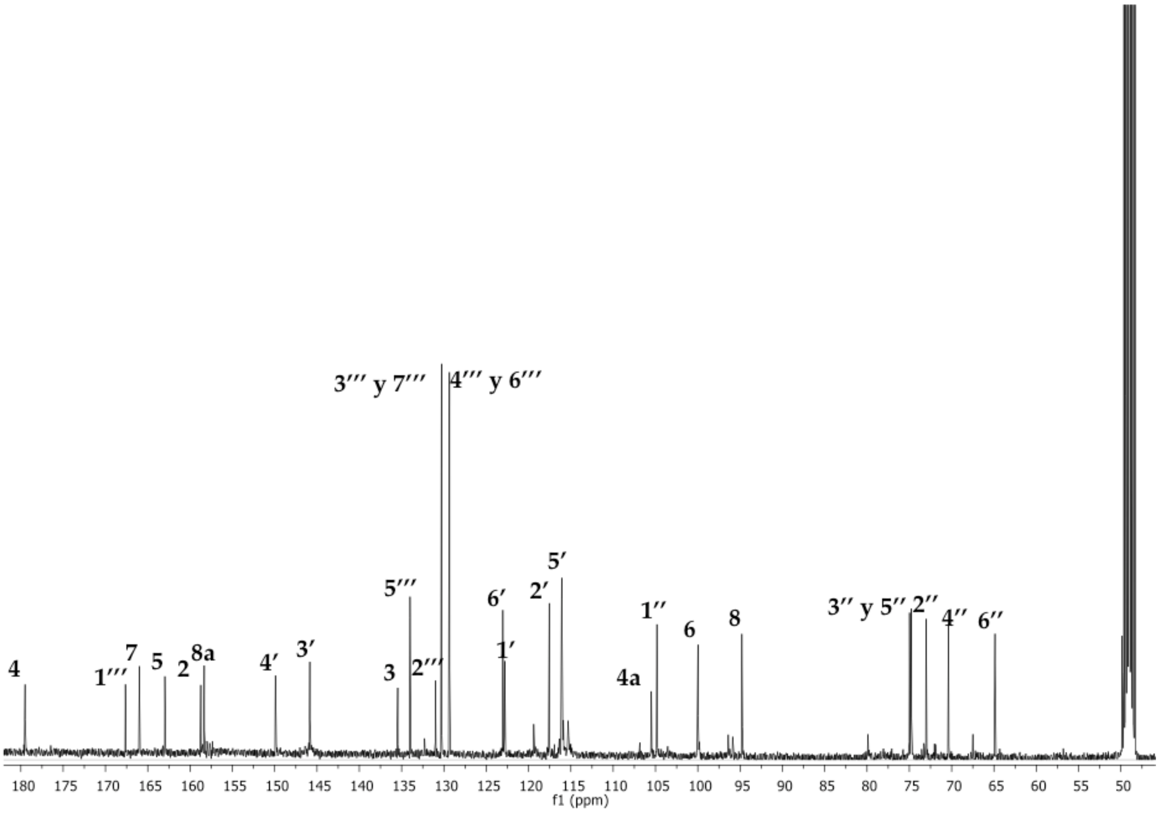

Figure S8. ^{13}C-NMR spectra of quercetin-3-*O*-(6″-benzoyl)-β-galactoside (**6**) (100 Hz, CH_3OH-d_4).

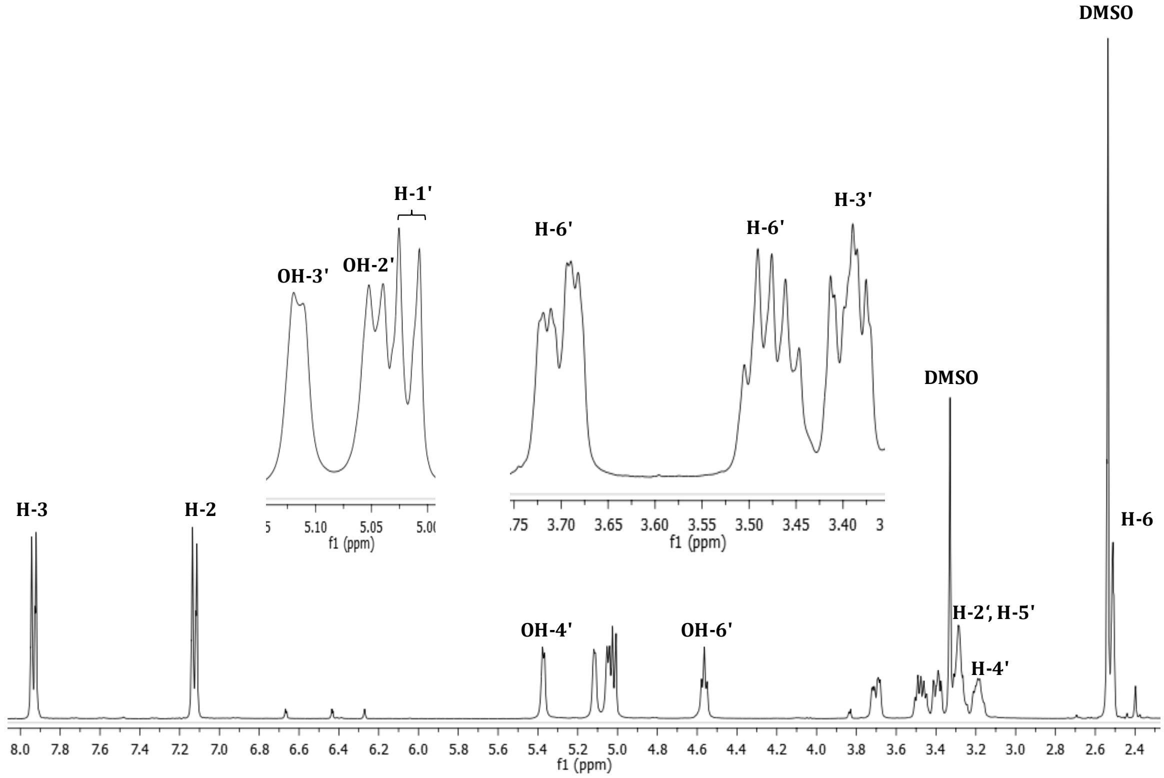

Figure S9. ^{1}H-NMR spectra of picein (**7**) (400 Hz, CH_3OH-d_4).

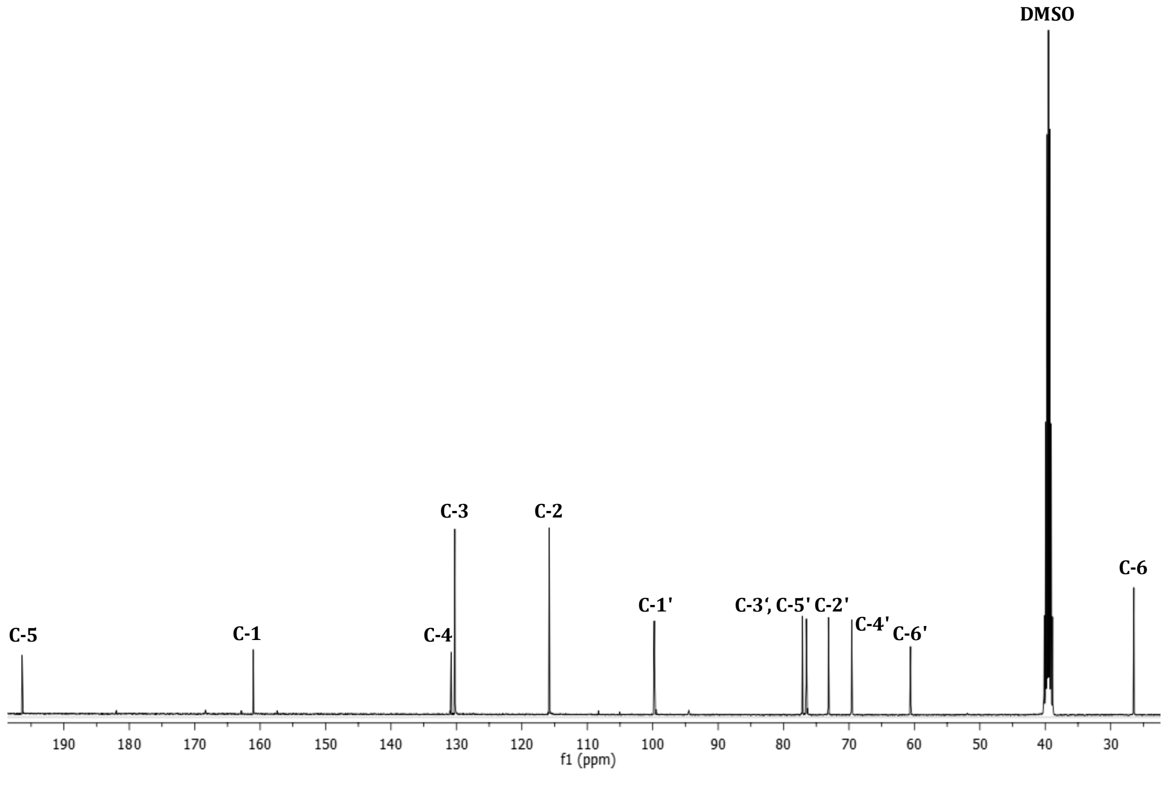

Figure S10. ^{13}C-NMR spectra of picein (**7**) (100 Hz, CH_3OH-d_4).

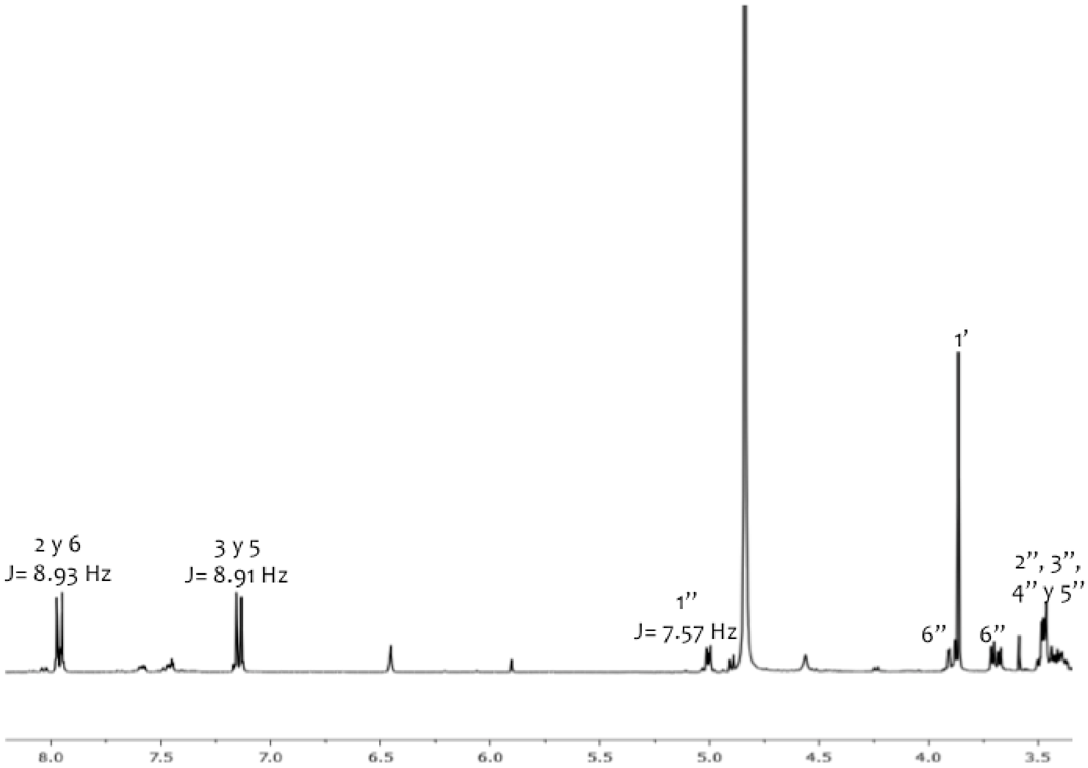

Figure S11. ^{1}H-NMR spectra of methylarbutin (**8**) (400 Hz, CH_3OH-d_4).

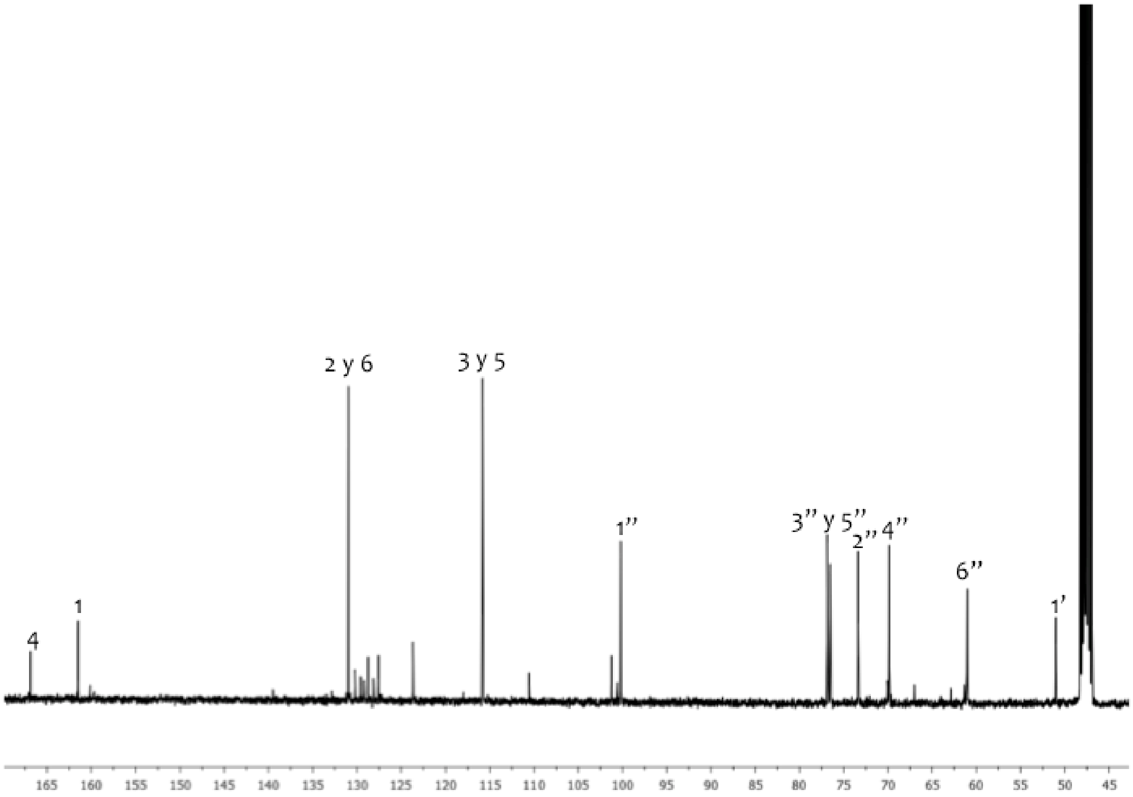

Figure S12. ^{13}C-NMR spectra of methylarbutin (**8**) (100 Hz, CH_3OH-d_4).

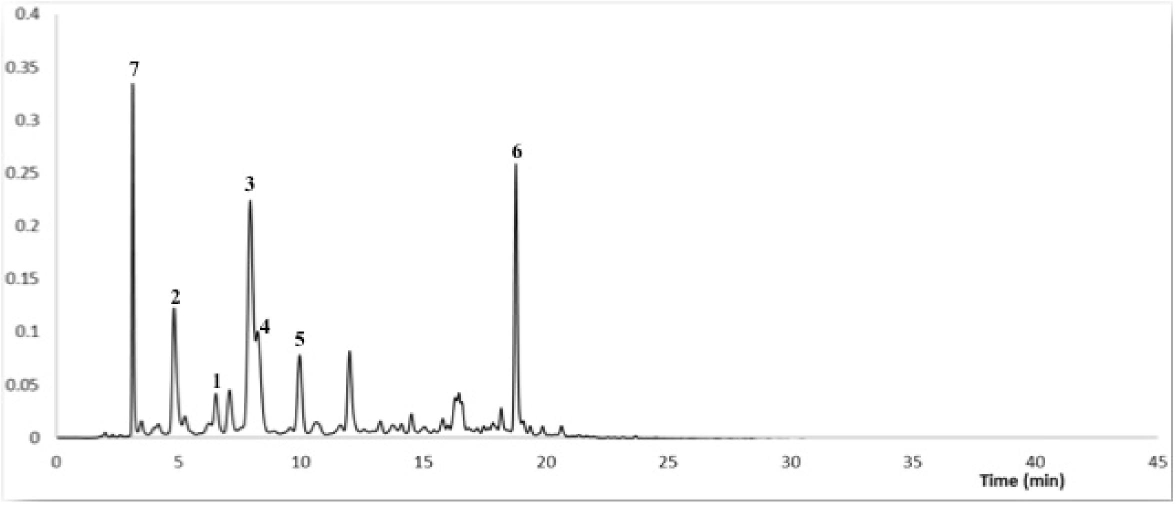

Figure S13. Chromatography profile of infusion of *V. corymbosa*. The chromatographic profile of AE was obtained by reverse-phase HPLC; Column: Symmetry C18; Sample: 100 µL of AE (1 mg/mL); Detection: 254 nm.

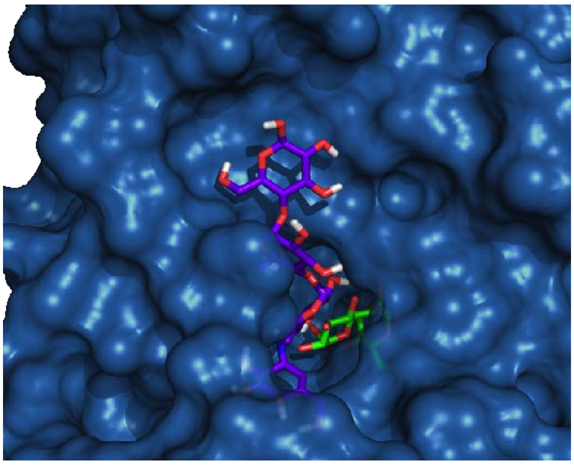

Figure S14. Structural model of the complex isomaltase (**green sticks**) with α-glucosidase and acarbose (**purple sticks**).

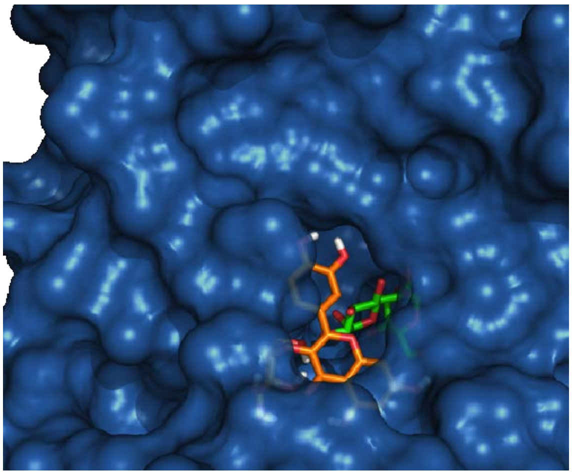

Figure S15. Structural model of the complex isomaltase (**green sticks**) with α-glucosidase and compound **4** (**orange sticks**).

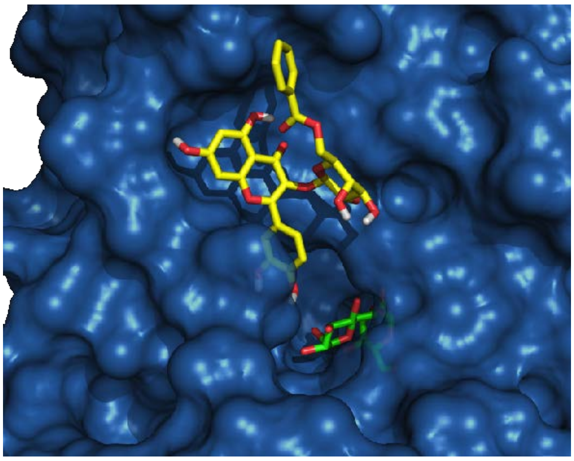

Figure S16. Structural model of the complex isomaltase (**green sticks**) with α-glucosidase and compound **6** (**yellow sticks**).
